# Calcium increases titin N2A binding to F-actin and regulated thin filaments

**DOI:** 10.1038/s41598-018-32952-8

**Published:** 2018-10-01

**Authors:** Samrat Dutta, Christopher Tsiros, Sai Lavanyaa Sundar, Humra Athar, Jeffrey Moore, Brent Nelson, Matthew J. Gage, Kiisa Nishikawa

**Affiliations:** 1 Center for Bioengineering Innovation, Northern Arizona University, Flagstaff, AZ 86011-4185 USA; 2 Chemistry Department, University of Massachusetts at Lowell, Lowell, MA 01854 USA; 3 Biological Sciences Department, University of Massachusetts at Lowell, Lowell, MA 01854 USA; 4 Mechanical Engineering Department, Northern Arizona University, Flagstaff, AZ 86011-15600 USA

## Abstract

Mutations in titin are responsible for many cardiac and muscle diseases, yet the underlying mechanisms remain largely unexplained. Numerous studies have established roles for titin in muscle function, and Ca^2+^-dependent interactions between titin and actin have been suggested to play a role in muscle contraction. The present study used co-sedimentation assays, dynamic force spectroscopy (DFS), and *in vitro* motility (IVM) assays to determine whether the N2A region of titin, overlooked in previous studies, interacts with actin in the presence of Ca^2+^. Co-sedimentation demonstrated that N2A – F-actin binding increases with increasing protein and Ca^2+^ concentration, DFS demonstrated increased rupture forces and decreased k_off_ in the presence of Ca^2+^, and IVM demonstrated a Ca^2+^-dependent reduction in motility of F-actin and reconstituted thin filaments in the presence of N2A. These results indicate that Ca^2+^ increases the strength and stability of N2A – actin interactions, supporting the hypothesis that titin plays a regulatory role in muscle contraction. The results further support a model in which N2A – actin binding in active muscle increases titin stiffness, and that impairment of this mechanism contributes to the phenotype in muscular dystrophy with myositis. Future studies are required to determine whether titin – actin binding occurs in skeletal muscle sarcomeres *in vivo*.

## Introduction

Titinopathies, inherited diseases caused by mutations in the titin (*TTN*) gene, are responsible for diverse cardiac and muscle diseases^1^. In the heart, titin mutations are linked to numerous forms of cardiomyopathy^2,3^. In skeletal muscles, titin mutations are associated with a rapidly growing number of disorders ranging from early onset diseases characterized by muscle degeneration and early death to late onset diseases that affect a small set of muscles after years of apparently normal function^1^. The underlying mechanisms that produce degenerative phenotypes in titinopathies remain largely unexplained^1^.

Since the discovery of titin^4^, many studies have investigated its interactions with other sarcomeric proteins using several different techniques. These studies revealed a variety of interactions in the M-line and Z-disk which highlight the important role of titin as a sarcomeric scaffold for myofibrillogenesis^5^. In the Z-disc, titin Z repeats interact with F-actin, alpha-actinin, and other proteins including telethonin. In the M-line, titin FnIII domains bind to the tail portion of myosin, as well as to MyBP-C and myomesin. Numerous studies have established roles for titin in myofibrillar assembly^6^, myofibrillar passive tension^7,8^, mechanosensing^9^, and hypertrophic signaling^10^.

In addition to these established roles for titin, Ca^2+^-dependent interactions between titin and actin have long been suggested to play a role in muscle contraction. Experimental evidence supports the existence of Ca^2+^-dependent interactions between I-band titin and actin. Using *in vitro* motility and binding assays, Kellermayer and Granzier^11^ found that a T2 fragment of titin (comprising the N2-line to M-line) from rabbit longissimus dorsi interacts with actin filaments and reconstituted thin filaments at pCa < 6.0. Although such interactions have been suggested to increase titin stiffness in active skeletal muscle^12–14^, this model has been slow to gain wide-spread acceptance^15^.

Despite significant efforts, subsequent attempts to discover Ca^2+^-dependent interactions between I-band titin and actin have been largely unsuccessful. Several studies investigated interactions between PEVK titin and actin using constructs based on human soleus (GenBank X90569^16^) and human cardiac titin (GenBank X90568^16^). Titin constructs based on skeletal muscle titin (GenBank X90569, residues 5618–7791) exhibited only Ca^2+^-independent interactions with actin^17–20^, and constructs based on cardiac titin (GenBank X90568) were inhibited by S100A1 when Ca^2+^ was present^20^. Only one study^21^ examined interactions between N2A titin (X90569, residues 5510–5507, Ig80-IS-Ig81-Ig82) and actin. Using co-sedimentation and immunofluorescence microscopy, this study found no interactions between titin and F-actin or reconstituted thin filaments outside the Z-disc. Notably, however, the terminal Ig83 domain was not included in this construct^21^.

Analysis of previous studies that investigated interactions between titin constructs and actin demonstrates that no previous studies included a sequence of 115 amino acids (residues 5508–5617 from X90569) at the N2A-PEVK intersection in titin, spanning most of Ig83 and the first 30 amino acids of the proximal PEVK region. Therefore, the goal of the present study was to test the hypothesis that amino acid residues in the N2A region of titin interact with actin filaments in the presence of Ca^2+^. We hypothesized that this previously unexamined region of N2A titin could be responsible for the Ca^2+^-dependent interactions with actin and reconstituted thin filaments observed previously by Kellermayer & Granzier^11^. Additional motivation came from the observations that: 1) an 83 amino acid deletion at the N2A-PEVK intersection in muscular dystrophy with myositis (*mdm*) mice leads to early muscle degeneration and death^22^; and 2) the *mdm* mutation prevents the increase in titin stiffness that normally occurs upon activation of wild type muscles, perhaps by disrupting titin-actin interactions^23^. In the present study, we used co-sedimentation, *in vitro* motility assays (IVM), and dynamic force spectroscopy (DFS) to characterize interactions between an N2A titin construct (Ig80-IS-Ig81-Ig82-Ig83) and F-actin or reconstituted thin filaments in the presence and absence and Ca^2+^.

## Methods

### Expression and Purification of N2A construct

**Figure 1:**
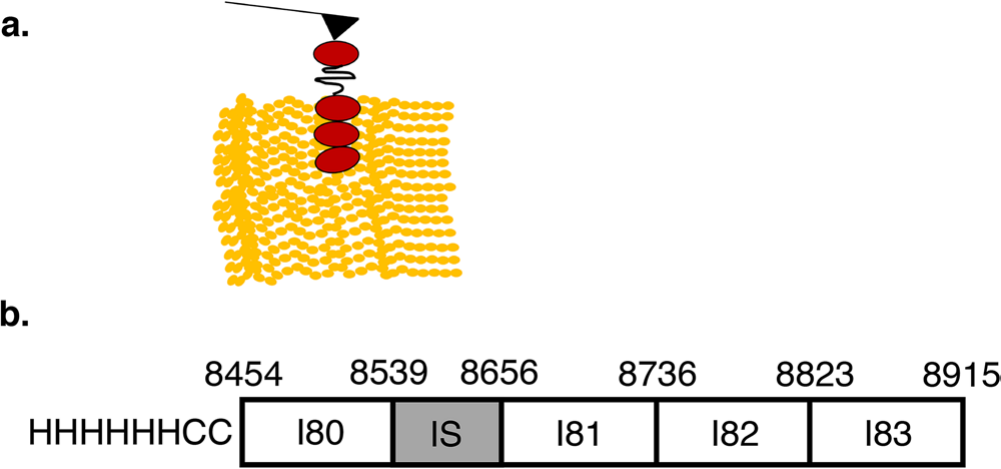
(**a**) Schematic of experimental setup showing N2A construct (Ig domains in red) attached to the AFM tip interacting with F-actin paracrystals on the surface. (**b**) Schematic of the N2A clone used in these studies. The position of the HIS-tag (HHHHHH) and cysteine residues (CC) are shown at the N-terminus. Numbers at the domain boundaries indicate the amino acid sequence of the clone based on annotation of NP_035782 in the NCBI database.

An N2A construct (Fig. 1) containing an N-terminal HIS-tag and two N-terminal cysteine residues was synthesized and subcloned into a pET 100/D plasmid (GeneArt, Biomatik, Regensburg, Germany). Expression was performed in BL21 (DE3) cells using auto-inducing media^24,25^ and overnight incubation at 30 °C with shaking at 300 rpm. Expressed N2A protein was found in the inclusion body. Induced cells were harvested and re-suspended in PBS with lysozyme, DNase, MgCl_2_, Triton X-100 and protease inhibitors. Re-suspended cells were freeze-thawed and sonicated. The inclusion body was harvested by centrifugation and the process was repeated three times, including 0.375 M urea in the third wash to remove non-specifically associated proteins. Following centrifugation, the pellet was re-suspended in chromatography buffer (20 mM Tris, 150 mM NaCl, 25 mM imidazole, 0.1% Tween-20). N2A protein was released from the inclusion body using a French press at 20,000 PSI with a 5 min hold before collection of lysate. Remaining insoluble protein was removed by centrifugation and N2A protein was loaded onto a HIS-Trap column (GE Healthcare) and eluted using 150 mM imidazole. Purity was assessed using Coomassie stained SDS-PAGE gels and protein concentration was determined using Bradford assays. Purified protein was stored at −20 °C in 50% glycerol.

### Actin Co-Sedimentation Assay

The N2A construct and F-actin were diluted in 25X reaction buffer (RB: 2.5 mM ATP, 5 mM DTT, 2.5 mM CaCl_2_, 25 mM MgCl_2_, 2.5 M NaCl and 50 mM Tris, pH 8) to a final concentration of 1X and 2 µM F-actin was mixed with three concentrations of N2A (2, 3, or 5 µM) in the presence of Ca^2+^ (pCa = 4). The assays were repeated in the absence of Ca^2+^ (pCa = 10) as a negative control for N2A binding. Ca^2+^-free samples were created by adding 1 mM EGTA (final concentration). Co-sedimentation assays were repeated three times for each combination of [Ca^2+^] and [N2A] for a total of 18 samples. Samples were incubated for 1 hr at 4 °C and F-actin was sedimented by centrifugation at 30,000 *g* for 30–45 min. Pelleted F-actin was washed with 1X RB to remove non-specifically bound protein and pelleted F-actin was solubilized using 8 M urea + 1 mM β-ME for >2.5 hours. Sample volumes were equalized using 1X RB and samples were separated on a 10% SDS-PAGE gel. Densitometry was performed using the rolling-ball algorithm on ImageJ. Two-way analysis of variance (ANOVA) was used to determine significance of effects of [Ca^2+^], [N2A], and their interaction on titin-actin binding as estimated from gel densitometry (α = 0.05).

### Titin attachment to AFM tip

N2A constructs were attached to AFM tips (Fig. 1) using standard procedures for thiol-maleimide crosslinking^26,27^. Silicon nitride AFM tips (MSNL, Bruker Inc., k = 30–40 pN/nm) were amino (APTES) functionalized following a previously published protocol^28^. Briefly, tips were treated with 30 µL of APTES and 10 µL triethylamine-TEA solutions inside a desiccator for 2 hours. After amino-functionalization, tips were treated with N-γ-maleimidobutyryl-oxysuccinimide ester before attachment. AFM tips were incubated overnight with thiol de-protected N2A constructs (7 nM) in PBS buffer (1X, pH 7.4) inside a humid chamber at 4 °C. Cross-linking was achieved through covalent-bond formation between the sulfhydryl groups present in the two N-terminal cysteines on the N2A-construct and the maleimide functionalized AFM tip.

### Polymerization and deposition of F-actin on lipid bilayer surface

**Figure 2:**
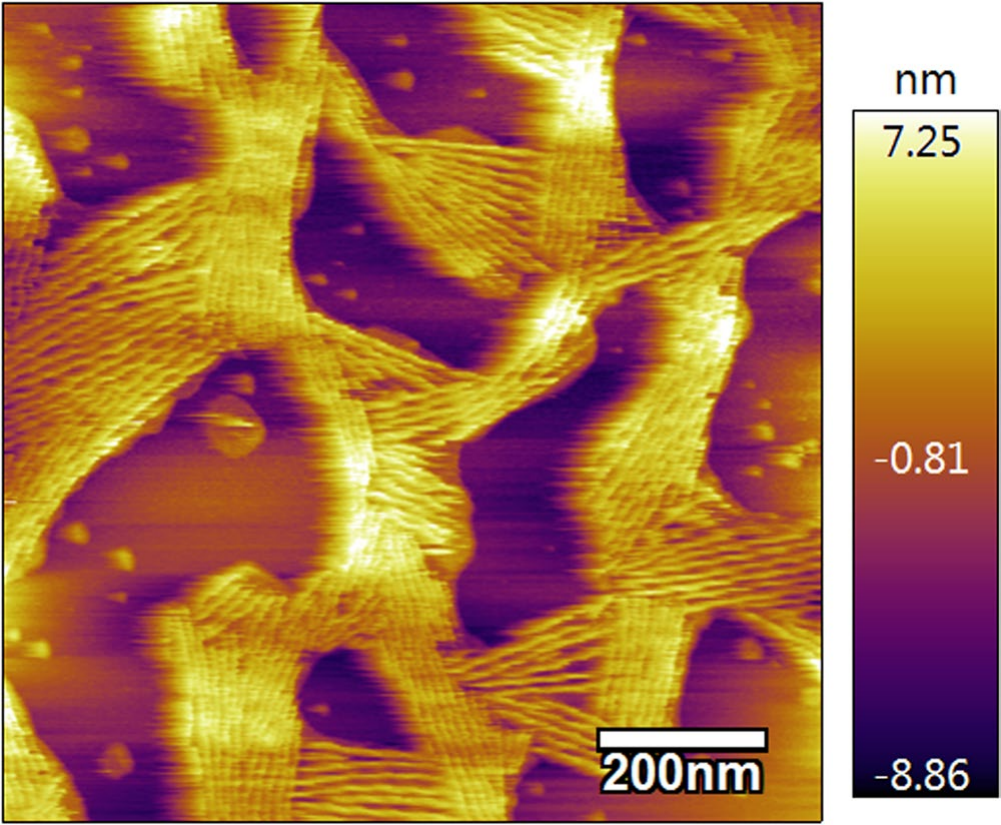
Tapping mode AFM image of F-actin paracrystals on charged lipid bilayer surface.

To obtain small unilamellar lipid vesicles, a 1:1 mix of dried 1,2-dipalmitoyl-sn-glycero-3-phosphocholine and 1,2-dipalmitoyl-sn-glycero-3-ethylphosphocholine was hydrated in buffer (10 mM Tris, 100 mM NaCl, 3 mM CaCl_2_, pH 7.4) at 1 mM final lipid concentration, followed by sonication for 10 min. A charged lipid bilayer was formed on a freshly cleaved mica surface by incubating at 55 °C for 20 min in a hydrated environment^29^. On the day of each experiment, globular actin (Cytoskeleton, Inc.) was polymerized to F-actin in buffer (5 mM Tris, 1 mM ATP, 0.5 mM CaCl_2_, 0.5 mM DTT, pH 8.0). F-actin was attached to the lipid bilayer surface by incubating for 30 min and stability was verified using AFM imaging (Fig. 2). F-actin polymerized using this approach was stable for ~3 hours, consistent with previously published results^29^, avoiding use of non-physiological stabilizing factors, such as phalloidin, that may affect molecular interactions.

### Single molecule force spectroscopy (SMFS) and dynamic force spectroscopy (DFS)

SMFS and DFS experiments enable direct measurement of molecular interaction forces between N2A constructs and F-actin. All SMFS and DFS experiments were performed using an Asylum atomic force microscope (MFP3D-infinity, Oxford Instruments, Santa Barbara, CA, USA) and silicon nitride cantilevers with stiffness of ~20–30 pN/nm. To verify the structure of N2A constructs, SMFS was performed by pulling the N2A constructs adsorbed on a mica surface in PBS (1X, pH 7.4) and unfolding behavior was observed. SMFS experiments were conducted in probing buffer (1X PBS, 1 mM ATP) in the presence (pCa = 4.0) or absence of Ca^2+^. Measurements in the absence of Ca^2+^ were performed by adding 700 µM EGTA (Sigma Aldrich) to the probing buffer to eliminate free Ca^2+^. During experiments, the AFM tip was brought in contact with the surface until a triggering force of 100 pN was applied and held for 0.5 s, allowing the N2A construct to interact with F-actin on the surface^26^. The tip was then retracted by 300 nm at constant, physiologically relevant velocities between 300–2000 nm/sec^17^. Probing was performed using multiple approach-retract cycles of the AFM tip over various locations on the lipid bilayer surface.

For each pulling velocity, ~100 force curves showing N2A-actin interactions, identified by polymer-like extension of the N2A constructs, were collected as described previously^26,27^. Each force curve was fit with the extensible worm-like chain (WLC) model to estimate contour length (L_c_), persistence length (L_p_), and rupture force (F), with L_c_ and L_p_ as free parameters. Because the insertion sequence allows the construct to behave like a tether, the loading rate could not be determined directly from the experimental data and the apparent loading rate (ALR) for each curve was instead calculated using Eq. 1 ^30^:

1 $$\frac{1}{r}=\frac{1}{({k}_{sp}v)}(1+\frac{{k}_{sp}{L}_{c}}{4}\sqrt{\frac{{k}_{B}{T}_{p}}{{L}_{P}{F}^{3}}})$$

where r is ALR, k_sp_ is the spring constant of the cantilever, v is the pulling speed, L_c_ is the contour length, k_B_ is the Boltzmann constant, T is room temperature (298 K), L_p_ is the persistence length, and F is the rupture force. Rupture force values for each experiment were measured, and histograms of rupture force distributions for N2A - F-actin interactions in the absence and presence of Ca^2+^ were plotted. For a rupture force distribution at a given apparent loading rate, the most probable rupture force was calculated by fitting the distribution with a probability density function (PDF)^26,27,31–33^:

2 $$p(F)={k}_{off}\exp (\frac{F{x}_{\beta }}{{k}_{B}T})\frac{1}{r}exp(-{k}_{off}{\int }_{0}^{F}exp(\frac{F{x}_{\beta }}{{k}_{B}T})\frac{1}{r}df)$$

where k_off_ is the off-rate constant and x_β_ is the distance of the transition state from the bound state. While k_off_ and x_β_ can theoretically be fit using Eq. 2, a more accurate fit is gained by measuring rupture forces at multiple loading rates. The relationship between rupture force and apparent loading rate is given by Eq. 3 ^34^:

3 $$F=\frac{{k}_{B}T}{{x}_{\beta }}ln(\frac{r{x}_{\beta }}{{K}_{off}{k}_{B}T})$$

Plotting values of rupture forces corresponding to maxima of PDF distributions against the logarithm of mean apparent loading rate allowed estimation of k_off_ and x_β_ from linear fits. The most probable rupture forces in the presence and absence of Ca^2+^ were compared using two-tailed T-tests (α = 0.05), and effects of ALR and [Ca^2+^] on k_off_ were tested using analysis of covariance (ANCOVA, α = 0.05), with [Ca^2+^] as the main effect and ALR as the covariable.

### *In-Vitro* Actin Motility (IVM) Assay

IVM assays measure the velocity of actin filaments on a substrate of myosin^35^ and have been used previously to investigate interactions between titin fragments and actin or reconstituted thin filaments^11,36,37^. Addition of actin binding proteins induces a frictional load on the filaments^35^, resulting in reduced velocity. In this study, chicken skeletal myosin in myosin buffer (0.3 M KCl, 25 nM BES, 5 mM EDTA, 4 mM MgCl_2_ and 0.1 M DTT) was mixed with F-actin in actin buffer (55 mM KCL, 25 nM BES, 5 mM EDTA, 4 mM MgCl_2_ and 0.1 M DTT) and 1 mM ATP. Samples were centrifuged at 30,000 *g* for 25 minutes to remove inactive myosin heads. Flow cells were prepared by treating sterilized cover glass with 1% nitrocellulose and attaching the cover glass to microscope slides using double-sided tape.

Myosin (235 nM) and N2A (475 nM) or BSA (475 nM) were incubated for 1 min in flow cells to allow proteins to bind to the nitrocellulose. Addition of BSA produced controls with the same ratio of myosin to protein in the presence and absence of N2A. Flow cells were washed with BSA (15 µM) in actin buffer to block unoccupied binding sites on nitrocellulose. Non-functional myosin heads were removed by adding 1 µM actin for 2 min and washing with actin buffer plus 1 mM ATP, followed by actin buffer. TRITC-labeled porcine cardiac actin (5 nM) in actin motility buffer (40 mM KCl, 25 nM BES, 5 mM EDTA, 4 mM MgCl_2_, 0.1 M DTT and 1 mM ATP in 0.25% methylcellulose) was added to the flow cell. As for the SMFS and DFS experiments, phalloidin was not used to stabilize the actin since fresh F-actin was prepared for each experiment and remained stable throughout the course of each experiment. Myosin was activated by addition of ATP and velocity of actin filaments was measured from images captured over 20 s using a Nikon Eclipse TE2000-u microscope with PTI IC-200 camera using XCAP Sequence Capture software. Images were captured at 3 frames/s and 16 bits per pixel, resulting in 60 frames for analysis. Filament velocity was determined using the MTrackJ plugin for Image J, which allows manual tracking of distance traveled by individual filaments over a specified time period.

Motility of fluorescently labeled F-actin was determined at pCa = 10.0, 8.0, 6.0 and 4.0 (n = 116–342 filaments per group, total = 1613 filaments), and repeated using reconstituted thin filaments at pCa = 10.0, 8.0, 7.5, 7.0, 6.5, 6.0, 5.0 and 4.0 (n = 116–408 filaments per group, total = 3441 filaments). Reconstituted thin filaments were generated by incubating F-actin with native rabbit skeletal muscle troponin (600 nM) and recombinantly expressed α-tropomyosin (600 nM) in actin buffer. Two-way ANOVA was used to determine significance of effects of treatment (N2A vs. BSA), [Ca^2+^], and their interaction on motility of F-actin and reconstituted thin filaments (α = 0.05). ANOVA was performed using the average filament velocity including stalled filaments. The data were fit to the Hill cooperativity equation using non-linear regression. Shapiro-Wilkes tests (α = 0.05) verified the assumption that the data were distributed normally (p = 0.8747 for BSA control and p = 0.7055 for N2A). V_max_, pCa_50_ and the Hill coefficient were determined for each condition (BSA vs. N2A).

## Results

We used three approaches to test the hypotheses that N2A titin binds to F-actin, and that Ca^2+^ increases the strength of this interaction. First, we used co-sedimentation assays to estimate the dissociation constant between N2A and actin under equilibrium conditions. Next, we used dynamic force spectroscopy (DFS) to directly measure the rupture forces and binding stability associated with dynamic interactions between single N2A and F-actin molecules. Finally, we used *in vitro* motility (IVM) assays to measure the effect of titin constructs on velocity of actin filaments and reconstituted thin filaments in the presence of myosin.

### N2A structural validation

**Figure 3:**
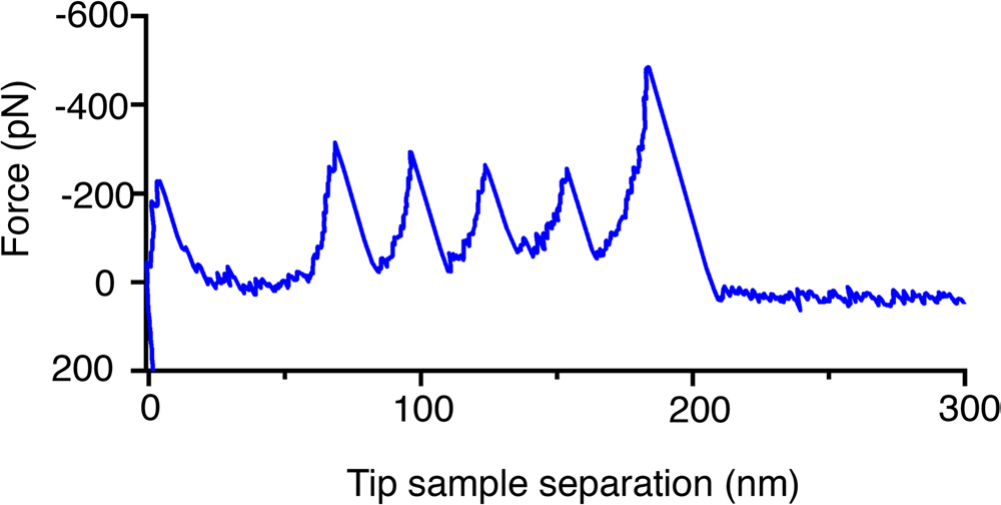
Force extension curve showing unfolding of all four Ig domains in the N2A construct.

The structure of N2A constructs was verified by obtaining force-extension curves using SMFS. When the AFM tip was retracted from the lipid bilayer surface, saw-tooth patterns corresponding to unfolding of each of the four Ig domains (Ig80, Ig81, Ig82 and Ig83) were observed (Fig. 3). The observed saw-tooth patterns exhibited L_c_ ~ 50 nm for the fully extended 117 amino acid insertion sequence (IS) and L_c_~30 nm for the Ig domains, verifying that the N2A constructs had the expected composition and structure.

### N2A-actin Co-sedimentation

**Figure 4:**
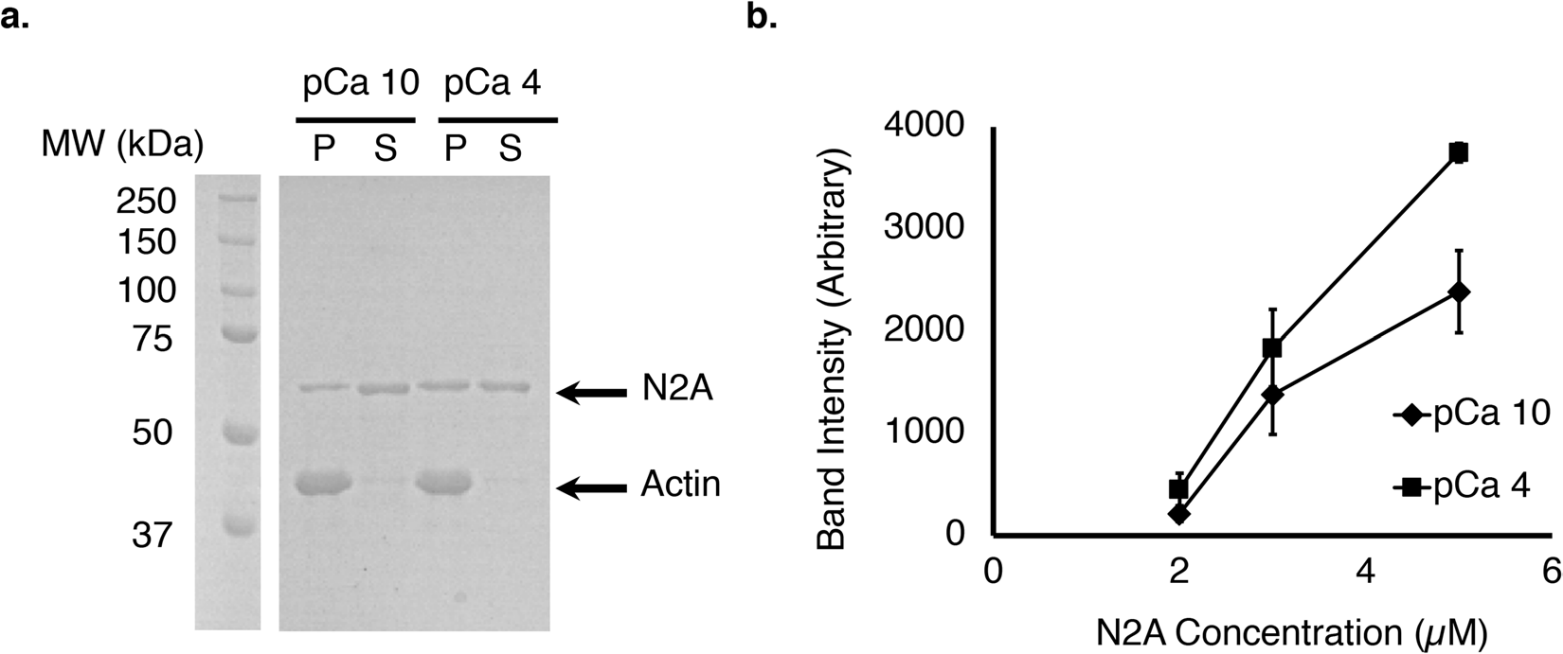
Ca^2+^ increases co-sedimentation of N2A constructs with actin filaments. (**a**) Example SDS-PAGE gel of 3 µM N2A bound to actin at pCa = 4 (squares) and pCa = 10 (diamonds). The gel was cropped to highlight the region containing the 3 µM N2A samples. The full-length gel is shown in Supplemental Figure S1. (**b**) Plot of average band intensities of bound vs. N2A concentration (N = 3 per group). The amount of bound [N2A] increases faster with N2A concentration at pCa = 4 than at pCa = 10 (ANCOVA, all p < 0.01).

In co-sedimentation assays, performed to estimate the dissociation constant for the interaction between the N2A construct and actin filaments, varying concentrations of N2A protein from 2–5 µM were incubated with F-actin at either pCa = 10 or pCa = 4. ANOVA demonstrated significant effects of [N2A] (p < 0.0001), [Ca^2+^] (p = 0.0003), and their interaction (p = 0.0125) on N2A – F-actin binding as estimated by densitometry. The amount of bound N2A protein increased as a function of N2A concentration (between 2–5 µM) and a fit of the data to estimate K_d_ also showed that K_d_ increases with Ca^2+^ concentration (K_d_ ~ 7 µM at pCa = 10 compared to K_d_ ~ 17 µM at pCa = 4; Fig. 4). While the small number of data points does not allow a definitive estimate of K_d_, the analysis shows that Ca^2+^ significantly increases binding of N2A to F-actin.

### Single molecule force spectroscopy (SMFS)

**Figure 5:**
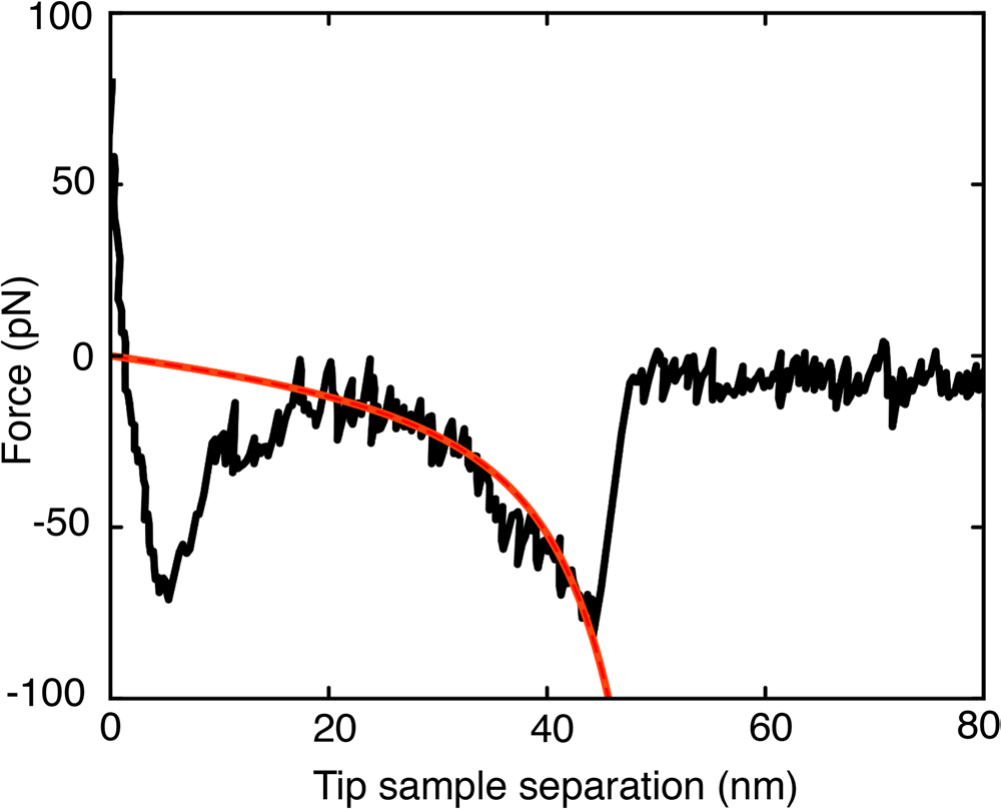
Force-extension curve (black) showing interaction between a single N2A construct and F-actin and fit to worm-like chain (WLC) model (red).

Direct measurements of single molecule interactions between N2A constructs and F-actin were made using SMFS. Retracting the AFM tip from the surface applied a mechanical force to the N2A - F-actin complex, producing a non-linear, polymer-like extension due to stretching of the N2A construct (Fig. 5). The construct detached from F-actin at rupture forces ranging from ~30–200 pN.

During retraction of the AFM probe, extension of IS and Ig domains (Fig. 5) was well approximated by the extensible WLC model (extension length/contour length >0.8), enabling determination of rupture force (F), persistence length (L_p_), and contour length (L_c_). L_p_ was 0.3 ± 0.15 nm in both the presence and absence of calcium, which is consistent with the persistence length value of an extended polymer reported elsewhere^37^. In control experiments performed in the presence and absence of Ca^2+^, when N2A constructs were probed against a charged lipid bilayer surface without F-actin, no polymer-like extension was observed and measured force values were in the range of instrument noise (10–15 pN). Similar results were obtained when a bare AFM tip with no N2A construct was probed against the actin functionalized lipid bilayer surface. These results confirm the specificity of the experimental approach in detecting N2A - F-actin interactions.

**Figure 6:**
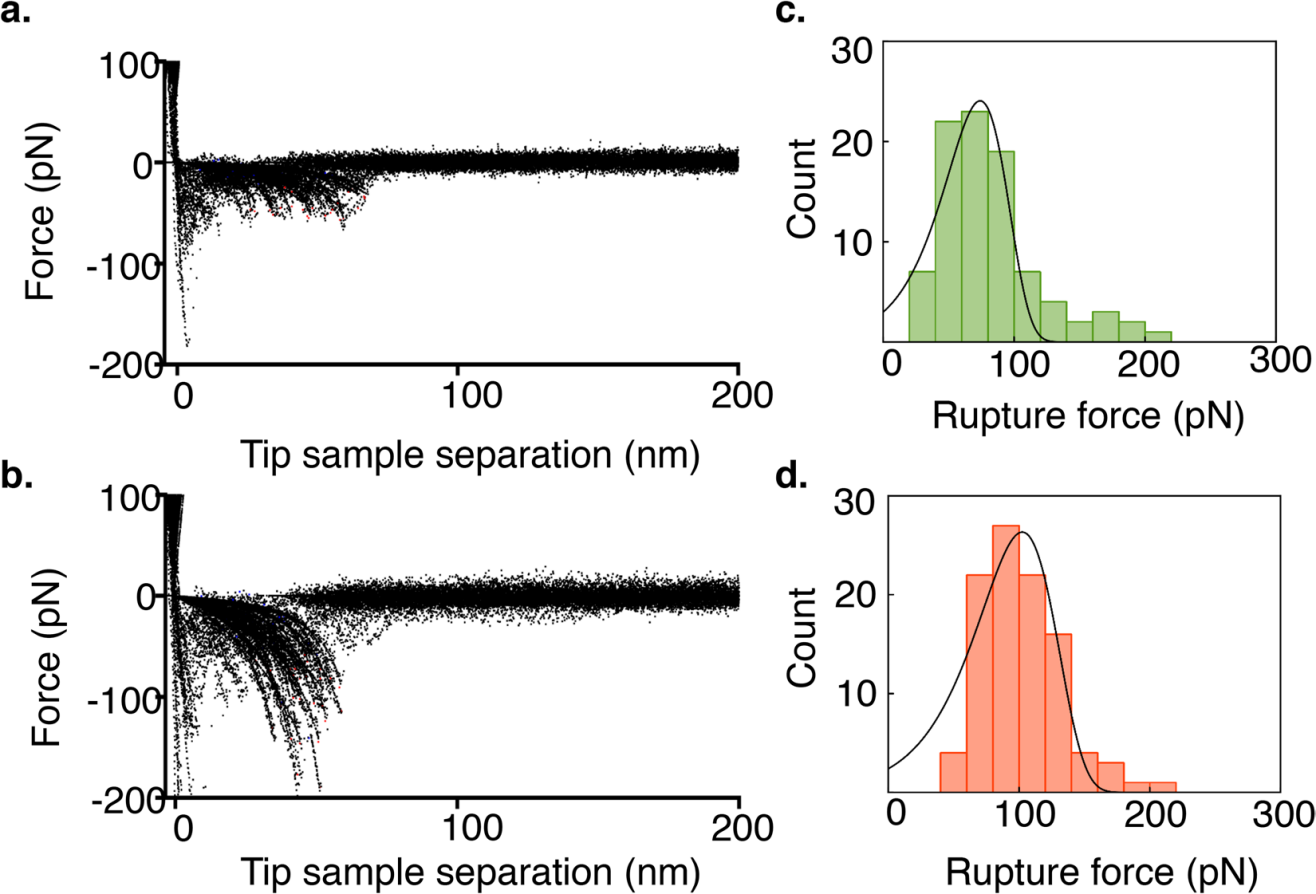
Ca^2+^ increases rupture forces of interacting N2A and F-actin molecules. Overlay of force-extension curves in the (**a**) absence and (**b**) presence of Ca^2+^. Histograms of N2A - F-actin rupture forces and most probable rupture force at a loading rate of 8000 pN/s in the (**c**) absence of Ca^2+^ (70 ± 40 pN) and (**d**) presence of Ca^2+^ (100 ± 40 pN).

Rupture events corresponding to dissociation of N2A - F-actin complexes were measured in the presence (pCa = 4) and absence of Ca^2+^ (Fig. 6a,b). The yield of interaction events was ∼5% in both cases. In the absence of Ca^2+^ (Fig. 6c), the maximum of the PDF was 70 ± 40 pN, while in the presence of Ca^2+^ (Fig. 6d), the maximum was 100 ± 40 pN (two tailed t-test, p < 0.001). This increase in rupture force demonstrates that Ca^2+^ significantly increases the strength of N2A - F-actin interactions.

**Figure 7:**
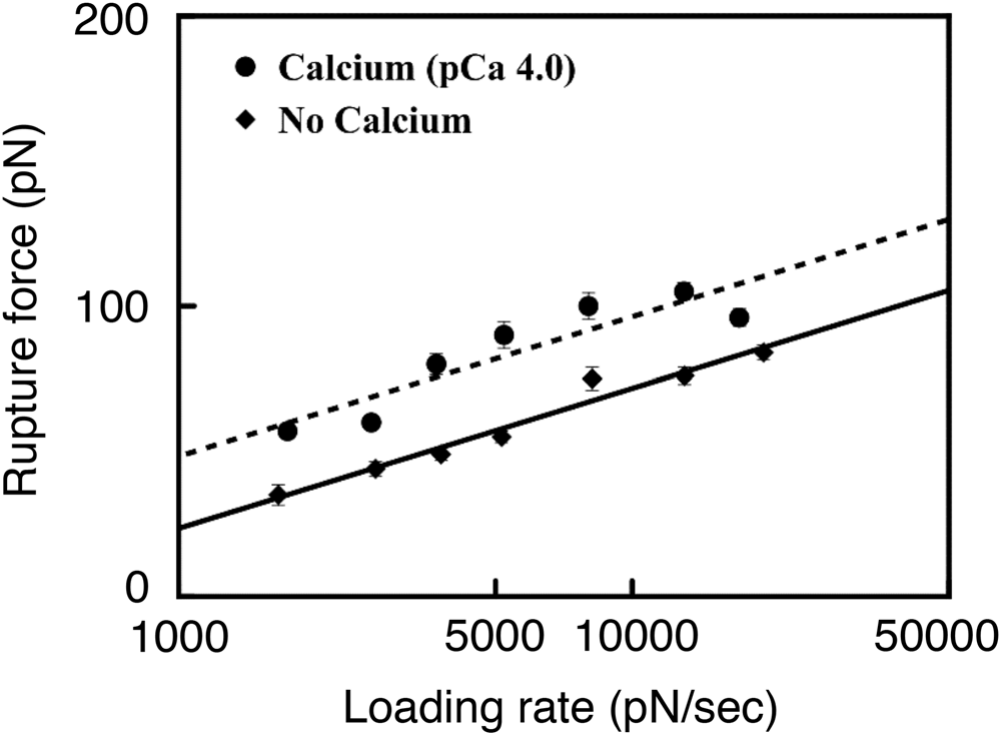
Dependence of N2A - F-actin rupture force on apparent loading rate (semi-log, in the absence (diamonds, k_off_ = 15.6 ± 2.7 s^−1^, x_β_ = 0.2 ± 0.01 nm) and presence (pCa = 4) of Ca^2+^ (circles, k_off_ = 4.7 ± 2.9 s^−1^, x_β_ = 0.2 ± 0.03 nm). Error bars represent standard error of the mean.

The stability of N2A - F-actin interactions in the presence (pCa = 4) and absence of Ca^2+^ was further characterized using dynamic force spectroscopy (DFS) by measuring the rupture force at various loading rates and using the Bell-Evans model^38^ to estimate k_off_ and x_β_ using Eq. 3. Because the N2A construct itself can stretch during loading due to the presence of the partially unstructured IS^39,40^, the apparent loading rate in Eq. 1 was calculated using L_c_ and L_p_ values for the stretched length of the construct that included the extended IS and Ig domains^26,31^. Both data sets were fit well using Eq. 3 (R^2^ = 0.9 for no calcium; R^2^ = 0.8 for pCa 4.0; P < 0.0001; Fig. 7), suggesting that the dissociation process undergoes a single barrier^38^. For the interaction between N2A and F-actin, the model yielded k_off_ = 15.6 ± 2.7 s^−1^ and x_β_ = 0.2 ± 0.01 nm in the absence of Ca^2+^, and k_off_ = 4.7 ± 2.9 s^−1^ and x_β_ = 0.2 ± 0.03 nm at pCa = 4. Thus, k_off_ decreased significantly in the presence (circles) vs. absence (diamonds) of Ca^2+^ (ANCOVA, p < 0.0001), indicating that Ca^2+^ increases the lifetime of N2A - F-actin interactions. The slope was not significantly different in the presence vs. absence of Ca^2+^ (ANCOVA, p = 0.7118).

### *In-Vitro* Motility Assays (IVM)

**Figure 8:**
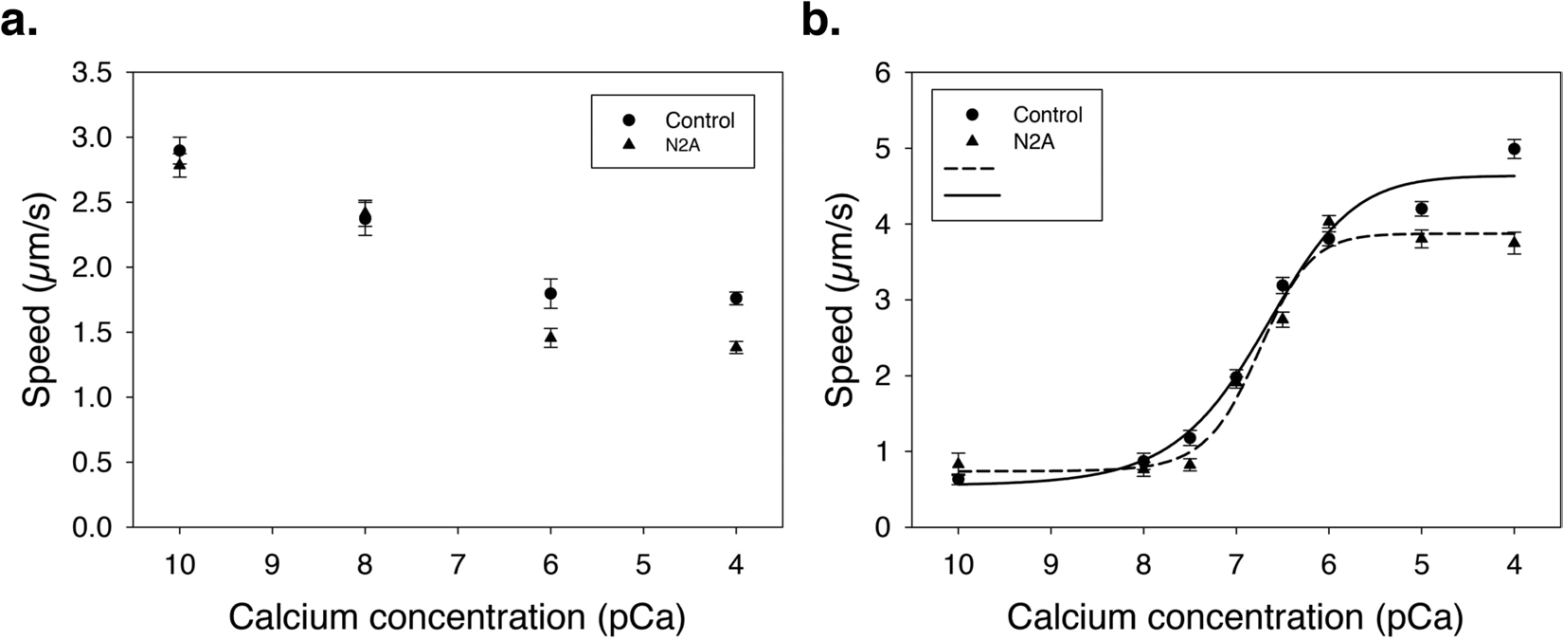
*In vitro* motility of F-actin and reconstituted thin filaments. (**A**) The N2A construct (triangles) decreases motility of F-actin in the presence of Ca^2+^ compared to BSA controls (circles). (**B**) *In vitro* motility of reconstituted thin filaments decreases in the presence of N2A at pCa = 4.0 and 5.0.

Co-sedimentation and force spectroscopy experiments demonstrate the ability of N2A to bind to F-actin or thin filaments. IVM was used to explore how the N2A constructs affect sliding velocity of F-actin and reconstituted thin filaments on a lawn of myosin. ANOVA showed that the velocity of F-actin decreased significantly in the presence of N2A vs. BSA (P < 0.0001) and also decreased (P < 0.0001) with increasing [Ca^2+^] (Fig. 8a). There was no difference in slope between N2A vs. BSA (P = 0.1268).

Similar experiments were conducted using regulated thin filaments, in which troponin and tropomyosin were present (Fig. 8b). The data were fit to the Hill cooperativity equation to determine V_max_, pCa_50_ and the Hill coefficient. Non-linear regression models were statistically significant for both BSA and N2A treatments (p ≤ 0.0007). V_max_, pCa_50_ and the Hill coefficients also explained a significant proportion of the variance in both groups (all p ≤ 0.0281). The pCa_50_ was unchanged in the presence of N2A (BSA control = 6.70, N2A = 6.76). The N2A-containing sample showed an increase in cooperativity based on the Hill coefficients (BSA control = 0.88, N2A = 1.564). V_max_ was ~1 µm/sec slower in the presence of N2A (3.13) compared to BSA control (4.09). There was little movement of reconstituted filaments at pCa 8 or 10, consistent with myosin binding sites being inaccessible due to the presence of troponin. ANOVA demonstrated significant effects of N2A vs. BSA (p < 0.0001), [Ca^2+^](p < 0.0001), and their interaction (p < 0.0001) on the velocity of regulated filaments. At pCa 4 and 5, there was a significant decrease in filament velocity in the presence of the N2A construct (Tukey’s HSD, p < 0.05).

## Discussion

Although many previous studies have investigated titin – actin interactions in muscle^17–20^, only Kellermayer and Granzier^11^ found Ca^2+^-dependent interactions between actin and the T2 fragment of titin, which extends from the N2-line to the M-line of muscle sarcomeres^41^. Using *in vitro* motility assays, they demonstrated that T2 reduced the velocity of F-actin and reconstituted thin filaments moving on a lawn of heavy meromyosin in the presence and absence of Ca^2+^. They further showed that Ca^2+^ (pCa < 6.0) further decreased actin motility. Titin – actin binding was confirmed using an *in vitro* immunofluorescence binding assay.

Since 1996, no studies have succeeded in replicating the results of Kellermayer & Granzier^11^ by showing Ca^2+^-dependent interactions between titin and actin. Various *in vitro* experiments demonstrated Ca^2+^-independent interactions between F-actin or reconstituted thin filaments and PEVK constructs based on skeletal muscle titin (GenBank X90569^17,19^). Furthermore, interactions between cardiac PEVK constructs (GenBank X90568) and actin are inhibited by the Ca^2+^-binding protein S100AI in the presence of Ca^2+^ ^20^. However, all previous investigations excluded a critical region of 115 amino acids in the N2A-PEVK border region of skeletal muscle titin, which notably includes 53 of the 83 amino acids deleted in *mdm*^22^. The results of the present study demonstrate Ca^2+^-dependent interactions between N2A titin constructs (Ig80-IS-Ig81-Ig82-Ig83) and F-actin or reconstituted thin filaments at pCa < 6.0, similar to the results of Kellermayer and Granzier^11^.

### Co-sedimentation and *in vitro* motility

Co-sedimentation experiments demonstrate that Ca^2+^ decreases the dissociation constant for the N2A – F-actin interaction. The small number of data points limit drawing conclusions about how the affinity changes as a function of Ca^2+^ beyond the demonstration that bound N2A increases with higher Ca^2+^ concentration (see Fig. 4), as also found by Kellermayer & Granzier^11^. Furthermore, the motility of both polymerized actin filaments (see Fig. 8a) and reconstituted thin filaments (see Fig. 8b) decreases in the presence of N2A in a Ca^2+^-dependent manner. These results support previous findings^11^ that the titin T2 fragment, which includes N2A and PEVK regions, reduced the velocity of actin filaments at pCa < 6. Previous studies observed that actin-binding proteins reduce the sliding velocity of actin on myosin, presumably because binding applies a frictional load to actin filaments, resulting in slower velocities^35^. Taken together, these studies suggest that N2A titin, specifically the Ig80-IS-Ig81-Ig82-Ig83 region, is responsible for binding of titin to actin in the presence and absence of Ca^2+^, and that titin-actin interactions are stronger when Ca^2+^ is present, as observed previously for the T2 fragment of titin^11^.

These results imply that N2A-actin binding is likely mediated by Ca^2+^ stabilization of actin binding sites in Ig domains. It is possible that Ca^2+^-dependent binding is mediated through Ig83. This model is consistent with the observed loss of function observed in *mdm* mice^23^, which have a partially deleted Ig83 domain^22^. This also would explain why titin-actin interactions were observed in our studies but were absent in Linke *et al*.^21^, as the constructs used in that study did not include Ig83. While stable binding might require additional Ig domains beyond Ig83, the existing data suggest that this region likely contains amino acids required for binding.

### Single molecule dynamic force spectroscopy

While co-sedimentation measures equilibrium binding, DFS experiments provide a direct measure of molecular interaction forces and off-rates between the proteins. As Kellermayer and Granzier^11^ observed for the T2 fragment, DFS also demonstrated that the N2A construct binds to F-actin in the presence and absence of Ca^2+^. Higher rupture forces in the presence of Ca^2+^ (see Fig. 6) indicate that the N2A-actin interaction is stabilized by Ca^2+^. The wide distribution of rupture forces in the presence and absence of Ca^2+^ observed in the present study implies a variety of molecular interactions. The most likely cause of this wide force distribution is random variation in the interaction due to stochastic thermal fluctuations and variable loading directions^42,43^. Other possible explanations include multiple N2A - F-actin interactions^44^ or multiple F-actin binding sites within each N2A molecule^27^, but both are unlikely. It is unlikely that the wide distribution is due to multiple N2A molecules interacting with F-actin because only force curves demonstrating single interaction events were included in the analysis. The force distribution in the absence of Ca^2+^ exhibits what may be a small second peak at approximately double the force of the primary peak, which could be caused by rare simultaneous rupture events. Because these events appear to be rare and at much higher forces, they are unlikely to contribute to the width of the distribution. Also, the first peak of the PDF (see Eq. 2) fit to the rupture force histogram should correspond to a single interaction^45^. That this force was significantly greater than the force observed with no actin present (15–20 pN) indicates that the measurement corresponds to interactions with actin rather than the lipid surface. Alternatively, if N2A constructs have multiple F-actin binding sites, then the rupture force histogram should exhibit multiple peaks corresponding to the interactions at each of these sites. Multiple peaks are not apparent in the rupture force distributions beyond the small second peak already noted (see Fig. 6c,d) making the presence of multiple binding sites unlikely, although the possibility that multiple peaks might emerge with a larger number of force curves cannot be definitively ruled out. The N2A-F-actin rupture length in both the presence and absence of Ca^2+^ showed wide variability, but some rupture length clustering was observed in the more pronounced rupture events in the presence of Ca^2+^. This result seems to point toward a specific actin binding location within the N2Aconstruct, but more experiments involving individual Ig domains are required to confirm this possibility.

The value of k_off_ for N2A – F-actin interactions was significantly lower in the presence of Ca^2+^, suggesting that Ca^2+^ increases not only rupture forces but also the stability of the interaction. When plotted as a function of the log of the ALR, the linear fit of rupture forces between N2A constructs and F-actin in the presence and absence of Ca^2+^ also suggests a one-dimensional dissociation energy landscape. For comparison, at comparable loading rates, the stability of the N2A - F-actin interaction measured here in the presence of Ca^2+^ (k_off_ ~ 4.7 sec^−1^) is an order of magnitude lower than the stability of Ca^2+^-independent skeletal PEVK interactions with F-actin (k_off_ ~ 0.4 sec^−1^)^17^. This difference could be due to several factors, including different actin binding sites on N2A and PEVK, as well as reduced entropic flexibility of actin filaments when stabilized by heavy meromyosin and phalloidin, as in their study. The higher conformational flexibility of our N2A construct combined with the dynamic nature of F-actin paracrystals formed on a lipid surface could reduce the stability of the N2A – F-actin interaction, as higher protein configurational entropy can reduce mechanical stability^46^.

Although single molecule force measurements can be difficult to interpret, a number of factors support the N2A – F-actin interactions observed in this study. Because F-actin and N2A constructs are polymers, their spatial arrangement is likely to influence how they interact under physiological conditions. In this experiment, the pulling direction of N2A constructs attached to the AFM tip was orthogonal to the axis of the F-actin filaments. Although this geometric arrangement may not be identical to how native N2A might interact with thin filaments under physiological conditions, covalent attachment of the N2A constructs to the AFM tip via N-terminal cysteines frees the entire N2A molecule to interact with F-actin. Additionally, because the loading direction of N2A constructs was perpendicular to actin filaments, the measurement was not affected by inter-connecting actin bonds being pulled at the same time, as would be the case if the pulling direction was parallel to the filament axis. Our simple experimental approach also requires no additional stabilizing factors, such as phalloidin, which might affect any measured interactions.

Control experiments demonstrated no interactions between N2A constructs and the charged lipid bilayer surface, and it is unlikely that measured forces could represent rupture of lipid bilayers or F-actin filaments. Puncturing a lipid bilayer using an AFM probe requires forces in the range of nN^47^, much larger than forces measured in the present study (~30–200 pN). Mechanically breaking actin filaments or paracrystals of actin filaments requires forces ~600 pN^17,48^, also much larger than those measured in this study.

### Role of N2A-F-actin interaction in muscle sarcomeres

Based on their observation that titin T2 fragments decreased *in vitro* motility of actin and thin filaments in the presence of heavy meromyosin (HMM), Kellermayer and Granzier^11^ hypothesized that, in addition to generation of passive tension, titin might play a regulatory role in muscle contraction. Recent reports using specific cleavage of titin in a transgenic mouse, with tobacco etch virus protease inserted into the distal Ig region, suggest that titin bears considerable force during isometric contraction^49^. Titin cleavage was observed to reduce isometric contractile force by 50% in homozygous muscle. Such large titin forces cannot occur without an increase in titin stiffness compared to passive muscle, in which straightening of proximal Ig domains exhibits very low effective stiffness. Recent experiments in which single myofibrils were stretched beyond overlap of the thick and thin filaments (where cross-bridges *per se* no longer contribute to force production), showed that titin stiffness increased by a factor of ~4 compared to passive myofibrils at the same length^12,13^. Further studies demonstrated that this increase in titin stiffness fails to occur in psoas muscles from *mdm* mice^23^, with a 779 bp deletion corresponding to 83 amino acids in the distal Ig83 and proximal PEVK regions^22,50^. Taken together, these results are consistent with a model in which binding of N2A titin to actin/thin filaments in active muscle increases titin stiffness by preventing elongation of proximal Ig domains at low force, as occurs during passive stretch^8^, and that this mechanism for Ca^2+^-dependent increase in titin stiffness is likely impaired by the *mdm* mutation.

Future studies are required to test whether titin-actin interactions as reported here *in vitro* limit extension of proximal Ig domains during active stretch of skeletal muscle sarcomeres. Granzier^51^ suggested that labeling of myofibrils with anti-titin antibodies could be used to directly observe extension of titin segments *in situ* during passive and active stretch. A recent study^52^ used the F146 antibody, which binds in the distal PEVK segment near the edge of the A-band, to investigate segmental titin elongation. They found evidence for an effect of Ca^2+^ but no evidence for actin interactions on extension of proximal and distal segments. It is important to point out, however, that the F146 antibody binds far from the N2A region and the proximal segment therefore includes most of the PEVK segment, making it relatively insensitive to changes in elongation of the N2A region. A more definitive test of whether N2A titin binds to actin in skeletal muscle sarcomeres requires use of antibodies that bind closer to the N2A region, either in the N2A region itself or between N2A and the Z-line.

## Electronic supplementary material

Supplementary information

## Data Availability

The datasets generated during the current study are available from the corresponding author on reasonable request.
