# Supplementary material for: Calcium increases titin N2A binding to F-actin and regulated thin filaments
Source: Sci Rep. 2018 Oct 1;8:14575. doi: 10.1038/s41598-018-32952-8 (PMC6167357; doi:10.1038/s41598-018-32952-8)
Supplement: Supplementary file 1 — Supplementary information [file 41598_2018_32952_MOESM1_ESM.docx]

Supplementary Information for:

**Calcium increases titin N2A binding to F-actin and regulated thin filaments**

Samrat Dutta^1^, Christopher Tsiros^2^, Sai Lavanyaa Sundar^3^, Humra Athar^2^, Jeffrey Moore^3^, Brent Nelson^4^, Matthew J. Gage^2^, and Kiisa Nishikawa^1*^

*Figure S1: Entire SDS-PAGE gel for the actin binding assay. The data for 3 µM N2A are also shown in Fig. 4. Samples of N2A construct at three concentrations (2 µM, 3 µM, and 5 µM) were co-sedimented with 2 µM F-actin in the presence (pCa = 4) and absence (pCa = 10) of Ca^2+^. The pellet (P) and supernatant (S) from the sedimentation experiments are shown for each experiment. The N2A and actin bands are highlighted by the arrows on the right side of the gel.*
